# Is there a role for [^18^F]-FMISO PET to guide dose adaptive radiotherapy in head and neck cancer? A review of the literature

**DOI:** 10.1007/s40336-023-00607-y

**Published:** 2024-01-23

**Authors:** Khrishanthne Sambasivan, Sally F Barrington, Steve EJ Connor, Timothy H Witney, Philip J Blower, Teresa Guerrero Urbano

**Affiliations:** 1 Department of Clinical Oncology, Guy’s and St Thomas’ NHS Foundation Trust, London, UK; School of Biomedical Engineering and Imaging Sciences, King’s College London, London, United Kingdom; 2 King’s College London and Guy’s and St Thomas’ PET Centre; School of Biomedical Engineering and Imaging Sciences, King’s College London, King’s Health Partners, London, UK; 3 Department of Neuroradiology, King’s College Hospital NHS Foundation Trust, London, UK Department of Radiology, Guy’s and St Thomas’ NHS Foundation Trust, London, UK; School of Biomedical Engineering and Imaging Sciences, St Thomas’ Hospital, King’s College London, London, UK; 4 King’s College London, School of Biomedical Engineering and Imaging Sciences, St Thomas’ Hospital, London, United Kingdom; 5 Department of Clinical Oncology, Guy’s and St Thomas’ NHS Foundation Trust, London, UK; Faculty of Dentistry, Oral & Craniofacial Sciences and School of Cancer & Pharmaceutical Sciences, King’s College London, London, United Kingdom

**Keywords:** [^18^F]FMISO, hypoxia, head and neck cancer

## Abstract

**Purpose:**

Hypoxia is a major cause of radioresistance in head and neck cancer (HNC), resulting in treatment failure and disease recurrence. ^18^F-fluoromisonidazole [^18^F]FMISO PET has been proposed as a means of localising intratumoural hypoxia in HNC so that radiotherapy can be specifically escalated in hypoxic regions. This concept may not be deliverable in routine clinical practice, however, given that [^18^F]FMISO PET is costly, time consuming and difficult to access. The aim of this review was to summarise clinical studies involving [^18^F]FMISO PET to ascertain whether it can be used to guide radiotherapy treatment in HNC.

**Methods:**

A comprehensive literature search was conducted on PubMed and Web of Science databases. Studies investigating [^18^F]FMISO PET in newly diagnosed HNC patients were considered eligible for review.

**Results:**

We found the following important results from our literature review:

1) Studies have focussed on comparing [^18^F]FMISO PET to other hypoxia biomarkers, but currently there is no evidence of a strong correlation between [^18^F]FMISO and these biomarkers.
2) The results of [^18^F]FMISO PET imaging are not necessarily repeatable, and the location of uptake may vary during treatment.
3) Tumour recurrences do not always occur within the pretreatment hypoxic volume on [^18^F]FMISO PET.
4) Dose modification studies using [^18^F]FMISO PET are in a pilot phase and so far, none have demonstrated the efficacy of radiotherapy dose painting according to [^18^F]FMISO uptake on PET.

**Conclusions:**

Our results suggest it is unlikely [^18^F]FMISO PET will be suitable for radiotherapy dose adaptation in HNC in a routine clinical setting. Part of the problem is that hypoxia is a dynamic phenomenon, and thus difficult to delineate on a single scan. Currently, it is anticipated that [^18^F]FMISO PET will remain useful within the research setting only.

## Introduction

Hypoxia has long been identified as a driver of resistance to radiotherapy, a major treatment modality in head and neck cancer (HNC). Positron emission tomography (PET) imaging of tumors using hypoxia-specific tracers is an attractive method of assessing hypoxia. PET is non-invasive, can be repeated, and provides spatial information across the whole tumor. The last point is especially relevant to locally advanced HNC, where it is feasible to deliver a dose escalation specifically to the hypoxic region identified within a tumour [1–3]; a concept known as ‘dose painting’. ^18^F-fluoromisonidazole ([^18^F]FMISO) is the most well-known and extensively investigated hypoxic tracer in HNC [4].

Numerous clinical studies have been conducted in HNC patients using [^18^F]FMISO PET imaging, evaluating different aspects, such as correlation with other hypoxia biomarkers [5–7], the ability to prognosticate in HNC patients [8], and the variation in uptake during serial imaging [9, 10]. Despite the wealth of published literature from these studies, [^18^F]FMISO PET has yet to become part of routine practice for HNC patients. A significant barrier to clinical adoption is expense and access to this tracer. In most countries, [^18^F]FMISO is not commercially available and is produced only at a handful of research institutions, making it costly to obtain [11]. Furthermore, its lipophilic nature leads to slow clearance of the tracer from blood/normal tissues which makes its use time consuming (as PET images need to be acquired 2 - 4 hours after tracer injection) and results in images with a low signal to background ratio. Finally, the many clinical studies on [^18^F]FMISO in HNC are heterogenous with regards to study design, image analysis (for example evaluating standardized uptake value (SUV)_max_ versus tumour-to-background ratio (TBR)) and research question. This lack of consensus makes it difficult to clearly understand where there is a role for [^18^F]FMISO PET in HNC patients.

Several studies have reported a prognostic association between [^18^F]FMISO uptake pretreatment and survival outcomes [8, 12–14]. A meta-analysis [15] of 4 trials that included a total of 120 HNC patients who had pretreatment [^18^F]FMISO imaging and subsequently received curative radiotherapy was recently reported. The patient populations varied in T stage, tumour volume and human papilloma virus (HPV) status. Nevertheless, a multivariate analysis (which included T stage and HPV status) found that baseline [^18^F]FMISO uptake had a significant impact on locoregional control (*p*=0.04) and overall survival (*p*<0.04) in HNC patients treated with radiotherapy. However, despite its role as a potential prognostic biomarker, [^18^F]FMISO PET has so far failed to transition into the clinic. Planning studies have shown that in theory, it is possible to increase the radiotherapy dose to [^18^F]FMISO hypoxic volumes without increasing normal tissue doses [16, 17]. Whether [^18^F]FMISO will progress into the clinic to routinely guide radiotherapy treatment for HNC patients remains to be seen. The justification and evidence to support radiotherapy dose escalation to [^18^F]FMISO PET based hypoxia need to be considered, along with the practicalities of conducting these scans.

The purpose of this narrative review is to summarize the key findings from clinical studies of [^18^F]FMISO PET in HNC patients so far, to help understand its utility and whether it could realistically be used to guide radiotherapy ‘dose painting’ in HNC. To achieve this, four key questions will be addressed:

1) Is there a surrogate and ‘easy to perform’ biomarker for the [^18^F]FMISO hypoxia phenotype on imaging? This would help select patients for [^18^F]FMISO imaging and avoid the cost and inefficiency of scanning all patients.
2) Is [^18^F]FMISO PET imaging reproducible and reliable?
3) Do locoregional recurrences occur within the initial hypoxic volume on [^18^F]FMISO PET, such that we could justify dose escalation to the hypoxic volume specifically?
4) What have we learned so far from dose escalation/de-escalation studies?

Of note, we have not included studies investigating the prognostic potential of [^18^F]FMISO PET, given this has been addressed in a meta-analysis [15].

## Methods

We performed a comprehensive search of Pubmed and Web of Science Databases up to 18 May 2023 to identify relevant articles published from 1992 onwards. The year 1992 was chosen as it is when the first-in-man imaging of [^18^F]FMISO was reported [18]. We searched for academic scientific papers whose abstracts included any of the following terms: ‘fluoromisonidazole’, ‘misonidazole’, ‘F-MISO’, ‘[^18^F]FMISO, ‘^18^F-MISO’ or ‘FMISO’. Studies were included which investigated [^18^F]FMISO PET imaging (either at baseline or during treatment) in newly diagnosed HNC patients. The articles were reviewed for applicability to our ‘key questions’ and overall, 40 publications were selected for inclusion. **Figure 1** shows the PRISMA flowchart for study selection.

Data were consistently recorded for each study and included the number of patients, [^18^F]FMISO imaging parameters, time course of [^18^F]FMISO scanning and the time point during treatment when scans were conducted. The key findings of the studies are summarized here as a narrative review as this was felt to be the most suitable format to provide an overarching view of [^18^F]FMISO in HNC.

## Results

A comprehensive literature review was undertaken and the details and focus of the resulting studies are illustrated in **Figure 2**. The majority (58%) were studies correlating [^18^F]FMISO PET either with tissue or blood hypoxic biomarkers, or other imaging biomarkers. **Figure 3** maps out the studies geographically. The 40 studies included in this review were performed at just 13 institutions, across 8 countries, and 18 of the published studies were from Germany alone.

The studies have been summarised in evidence tables (see Tables 1-8), with some entered twice if they addressed 2 topics (for example repeat imaging *and* correlation with ^18^F-fluorodeoxyglucose (^18^F-FDG)). The results are discussed in detail below, in line with our ‘key questions’.

### 1) Correlation of [^18^F]FMISOPET with other biomarkers – is there a surrogate?

[^18^F]FMISO PET scans are both expensive and time consuming to conduct (as images are acquired 2-4 hours after tracer injection), therefore an easily accessible ‘surrogate’ hypoxia biomarker, which could help select patients who would benefit from [^18^F]FMISO imaging, would be desirable. Alternative biomarkers include hypoxia associated protein immunohistochemistry (IHC), hypoxic gene expression, oxygen electrode measurement (all tumour based) and serum osteopontin. Since all patients undergo a biopsy and blood tests at diagnosis, it would be ideal if one of these investigations could be applied to select patients for [^18^F]FMISO PET imaging. The studies correlating [^18^F]FMISO PET with other hypoxia biomarkers are summarised in Tables 1 and 2.

### Studies correlating hypoxia protein immunohistochemistry with [^18^F]FMISO uptake

Immunohistochemical markers of hypoxia include hypoxia-inducible factor 1-alpha (HIF1α), carbonic anhydrase 9 (CaIX) and glucose transporter 1 (Glut-1). These are endogenous proteins whose expression is upregulated in hypoxic conditions [19] and can be measured using IHC on tumour biopsy specimens. **Table 1** outlines the six studies [6, 11, 20–23] which assessed the relationship between hypoxia on [^18^F]FMISO PET and IHC markers of hypoxia. Overall, the results are mixed, with three studies [6, 11, 20] reporting a positive correlation between hypoxia IHC markers and hypoxia on [^18^F]FMISO PET, and two studies [22, 23] concluding that there was no association. Nicolay et al [21] conducted the largest study, assessing the relationship with hypoxia IHC (both CaIX and HIF1α) and [^18^F]FMISO PET conducted at different time points (week 0, 2 and 5) in 49 patients undergoing chemoradiotherapy. There was no correlation between either HIF1a or CaIX expression and [^18^F]FMISO hypoxia in treatment-naïve patients. They did however find an association between hypoxia IHC and ‘adverse hypoxia dynamics’ on [^18^F]FMISO PET, i.e., delayed resolution of hypoxia on PET scans during radiotherapy treatment. A major issue with using tumour IHC is sampling bias. Hypoxia is typically heterogeneously distributed across a tumour, so a single biopsy sample may not be representative of the whole tumour.

It appears that there may be a relationship between HIF1α /CaIX/Glut-1 expression and [^18^F]FMISO imaging, but current evidence is insufficient to propose a proxy biomarker of [^18^F]FMISO uptake. This question could be answered more fully by using archival tumour samples from previous [^18^F]FMISO trials to assess correlation with HIF1α or CaIX. It should be noted, however, that the markers measure different aspects of hypoxia and may not correlate with each other. For example, HIF1α expression represents the transcriptional changes that occur in response to a chronically hypoxic tumour microenvironment whereas [^18^F]FMISO uptake is a direct indicator of intracellular hypoxia, both acute and chronic.

### Studies correlating other hypoxia biomarkers with [^18^F]FMISO uptake

The other hypoxia biomarkers which have been studied in relation to [^18^F]FMISO imaging are oxygen electrode measurements, gene signatures, and plasma hypoxia markers. The details of these studies are summarised in **Table 2**. Oxygen electrodes allow direct measurement of hypoxia by inserting small needles into tumours to measure the partial pressure of oxygen (pO_2_). Three studies [24–26] correlated pO_2_ readings with [^18^F]FMISO hypoxia (in a total of 58 patients) and all found a strong correlation. These results were useful in that they validated [^18^F]FMISO PET as a means of detecting hypoxia, but they do not provide a practical representative biomarker of [^18^F]FMISO hypoxia. Oxygen electrode measurement is an invasive procedure which requires directly accessible tumours, so it cannot be used in a routine clinical setting.

Hypoxic gene signatures [27–29] refer to a collection of genes whose expression is upregulated or downregulated in response to hypoxia. They can be measured from a tumour biopsy specimen and are thought to represent the hypoxic phenotype of the overall tumour. Signatures are able to prognosticate in HNC [30] and also predict the benefit of hypoxia modification therapy in HNC [31]. To date, there is only one published study [7] analysing the relationship between hypoxia gene expression and [^18^F]FMISO uptake in a cohort of 42 HNC patients treated with radiotherapy. Correlations were assessed at baseline and at different time points during radiotherapy. There was only a weak association between hypoxic gene signatures and [^18^F]FMISO uptake at baseline (r=0.20) which increased at weeks 1 (r=0.38) and 2 (r=0.43) during radiotherapy.

The final study [32] in **Table 2** investigated the association between [^18^F]FMISO PET imaging and plasma hypoxia markers (osteopontin, vascular endothelial growth factor (VEGF), galectin-3 and circulating tumour growth factor (CTGF)). The most promising result was obtained with serum osteopontin, a protein whose plasma concentration has been shown to increase in conditions of tumour hypoxia [32]. There was a moderate correlation between osteopontin levels and the baseline hypoxic volume (r=0.579), and residual hypoxia on [^18^F]FMISO PET imaging (p<0.05) during treatment. Of note, osteopontin has been shown to inversely correlate with pO_2_ in HNC and also to prognosticate and predict benefit from hypoxia modification therapy in HNC [33]. Given that it is easily obtained by a blood test, it could potentially be an ideal ‘screening’ biomarker to select patients who would benefit from [^18^F]FMISO PET, but further studies are required to validate this concept.

### Correlation of [^18^F]FMISO PET with other imaging modalities

#### Correlation with ^18^F-FDG PET

^18^F-FDG PET is a routine investigation for many newly diagnosed HNC patients and hence it is convenient to assess correlation with [^18^F]FMISO PET. ^18^F-FDG PET provides assessment of glycolysis in tissue, which is a process affected by hypoxia [34]. HIF1α (activated in areas of low oxygen) upregulates both glucose transporters (GLUTs) and glycolytic enzymes [35], and therefore it is conceivable that ^18^F-FDG PET could be a surrogate marker of hypoxia. **Table 3** details the studies correlating ^18^F-FDG and [^18^F]FMISO PET. The majority of these studies showed a weak to moderate correlation between the two imaging modalities [6, 11, 24, 26, 34, 36–38], one showing no association between ^18^F-FDG and [^18^F]FMISO PET [39], and only one a strong association (r=0.81) [40]. Two other studies [41, 42] that demonstrated a strong relationship used ‘second order’ features on ^18^F-FDG PET; one looked at ‘total lesion glycolysis’ (SUV_mean_ multiplied by metabolic tumour volume on ^18^F-FDG PET) and found a correlation coefficient of 0.85 with ‘total lesion hypoxia’ (SUV_mean_ multiplied by hypoxic volume) on [^18^F]FMISO PET. The other study [41], used a radiomics signature from the CT, and found that this, in combination with ^18^F-FDG PET, improved the ability to predict for hypoxia on [^18^F]FMISO PET (with an area under the curve (AUC) of 0.83). In contrast, Kroenke et al. [37] used ‘texture analysis’ of the tumour on ^18^F-FDG PET and found that this did not improve the ability of ^18^F-FDG PET to predict [^18^F]FMISO uptake.

Given the many studies conducted, all with varied outcomes, it seems unlikely that ^18^F-FDG PET can be used to select patients for [^18^F]FMISO imaging. Furthermore, although some studies were able to demonstrate a correlation between degree of ^18^F-FDG and [^18^F]FMISO uptake, there were instances of low [^18^F]FMISO /high ^18^F-FDG uptake [34] and vice versa, showing that ^18^F-FDG and [^18^F]FMISO PET provide complementary and separate information from each other. From a biological perspective this is not surprising, given that one relates mainly to tissue glucose consumption and the other to tissue hypoxia.

#### Correlation with MRI

In recent years, attention has turned to multiparametric MRI (mpMRI) and its ability to provide information about tissue perfusion (dynamic contrast enhanced/DCE-MRI), cellularity (diffusion weighted imaging/DWI-MRI), and oxygenation (transverse relaxation time/T_2_*MRI).

These are all processes central to the development of tumour hypoxia. The six studies which have compared mpMRI with [^18^F]FMISO PET are summarised in **Table 4**. Two studies [43, 44] identified a relationship between DCE-MRI and [^18^F]FMISO uptake, with reduced K_trans_ (a measure of perfusion) in the hypoxic volume. Data on ADC are conflicting, with both decreased [45] and increased [46, 47] values being reported in the hypoxic volume. Of these modalities, T_2_* MRI is the most direct marker of hypoxia, as it measures the concentration of deoxygenated haemoglobin. The study which compared T_2_* MRI to [^18^F]FMISO PET [40] did not find a correlation, which again can be explained by the fact that they measure different processes; blood oxygenation versus intracellular oxygenations. MRI has the benefit of higher resolution compared to PET and would be ideal to identify tumour hypoxia for radiotherapy planning, but currently we do not have sufficient evidence to propose it for this role.

### 2) Is [^18^F]FMISOimaging repeatable and reproducible?

#### Studies repeating [^18^F]FMISOPET at baseline

Three studies [48–50] repeated [^18^F]FMISO PET scans within a short time frame (2-3 days) of each other (without interval treatment) to assess repeatability. They are described in **Table 5**. *Okamoto et al* [49] demonstrated that [^18^F]FMISO imaging is highly repeatable and that the maximum uptake location of [^18^F]FMISO varied by only 4 mm between two repeat scans. In contrast *Lin* [48] and *Nehmeh et al* [50] reported similar hypoxic volumes in ~ 50% of patients, but a significant variation in the location of tumour hypoxia in the other half of patients. The proposed explanation is that [^18^F]FMISO captures both acute and chronic hypoxia [50], and so stable uptake may be representative of chronic hypoxia only. Although patient numbers in these studies were small (7-14), the findings lend caution to the concept of dose escalating to a hypoxic volume generated from a single [^18^F]FMISO PET study in a patient. This is well displayed in the study by *Lin et al* [48] (a dose planning study), which showed a decrease in the prescribed uniform dose to the hypoxic volume of up to 12 Gy, between two serials scans (which were separated by 3 days), due to instability of the hypoxic region.

#### Studies repeating [^18^F]FMISO PET during radiotherapy treatment

Several studies interrogated hypoxia dynamics during radiotherapy treatment and are displayed in **Table 6**. All the studies showed that in the majority of patients, reoxygenation occurs and the degree of hypoxia, measured by [^18^F]FMISO PET, reduces through the course of treatment. Interestingly, although several studies comment on ‘residual’ hypoxia during treatment, only two formally reported on the geographical stability of the hypoxic volume [51, 52]. *Bittner et al*. [51] found a 72% overlap of the hypoxic volume from week 0 to week 2, suggesting that ‘residual hypoxia’ is an appropriate term. In contrast, *Carles et al*. [52] looked at spatial variation of hypoxia and found that only 24% of patients had geographically ‘stable’ hypoxia throughout their treatment, and that these patients had a better prognosis in terms of locoregional control. These findings are hugely important, as they undermine the concept of dose painting to the hypoxic volume seen on a single baseline [^18^F]FMISO PET and suggest that systemic hypoxia modification therapy, or dose escalation to the whole GTV, may be more appropriate to compensate for spatiotemporal changes in hypoxia.

### 3) Where do locoregional recurrences occur in relation to the initial hypoxic volume?

Despite the wealth of published studies on [^18^F]FMISO PET in HNC, only four were identified which correlated recurrence patterns to the initial hypoxic volume (see **Table 7**). From these, one study [53] concluded that recurrences arise from the original hypoxic subvolumes, with a median overlap of 42% between recurrence volume and the initial hypoxic volume. Two studies [9, 54] showed that a significant proportion of recurrences (33-50%) occur outside the pretreatment hypoxic volume. *Nishikawa et al*. [55] analysed pretreatment [^18^F]FMISO PET images from 21 patients with nasopharyngeal carcinoma (of whom nine recurred) to generate a risk model. They found that within the imaged tumour region, voxels with [^18^F]FMISO tumour to muscle ratio (TMR) > 2.42 predicted a recurrence rate of 30% within the same voxel. The AUC for this prediction model was only 0.59 however, and the authors concluded that the predictive value of pretreatment [^18^F]FMISO PET was insufficient for up-front dose escalation to the regions with high uptake, i.e. the hypoxic volume.

Overall, these findings suggest that although hypoxia is a known cause of radioresistance, there is not enough evidence to suggest that recurrences arise from the hypoxic regions identified on pretreatment imaging, especially when determined from a single scan. It should be noted, however, that the recurrence data stem from a total of only 70 patients, across 4 studies. Further knowledge is therefore required on disease recurrence and its relation to hypoxic volumes.

### 4) What have we learned so far from dose modification studies?

Given that the hypoxic uptake on [^18^F]FMISO PET carries important prognostic information [15], trials are underway to determine if radiotherapy can be dose de-escalated for patients with a good prognosis (absence or early resolution of hypoxia) and conversely escalated for patients with hypoxic tumours. So far 3 dose modification trials using [^18^F]FMISO PET as a biomarker have been published; see **Table 8**.

#### Dose de-escalation studies

The first [^18^F]FMISO de-escalation study was published in 2016 [23] on 33 patients with human papilloma virus (HPV)-positive oropharyngeal cancer. The radiotherapy dose to the metastatic lymph nodes was reduced by 10 Gy to 60 Gy in patients who had resolution of hypoxia at week one of radiotherapy. Ten patients had their radiotherapy dose de-escalated and remained recurrence free at two years. The second de-escalation study [56] was in a cohort of 19 HPV-positive oropharyngeal cancer patients who were treated with resection of the primary tumour and radiotherapy to the nodes followed four months later by a neck dissection. The radiotherapy dose was reduced to 30 Gy in 15 patients who had no hypoxia at either pre- or intra-treatment [^18^F]FMISO PET imaging. Eleven of the 15 patients had a pathological complete response. As these were pilot studies, neither had a comparative cohort in which patients received radiotherapy dose de-escalation despite hypoxia on PET. Given that the tumours were HPV-positive, it is possible their outcomes would have still been favourable, despite the observed hypoxia on PET.

A larger scale de-escalation study (clinical trial identifier NCT03323463; n=300) at Memorial Sloane Kettering is currently underway using [^18^F]FMISO PET to select patients to receive a de-escalated dose of radiation (30 Gy) if no hypoxia is observed on pre/intra-treatment imaging. In this study, all patients will receive two cycles of concomitant chemotherapy and surgical resection is no longer mandatory. However, this trial will not determine if it is the absence of hypoxia (on [^18^F]FMISO PET) that renders the patients suitable for treatment de-escalation as no randomisation to standard treatment vs. de-escalation is planned.

#### Dose escalation trial

So far one randomized phase II study has been published [57], which looked at dose escalation to the hypoxic volume alone on [^18^F]FMISO PET. Patients with a hypoxic volume pretreatment were randomised to receive standard chemoRT (70Gy in 35 fractions) or escalation of up to 10% with 77 Gy to the hypoxic volume only. The trial closed prematurely due to slow accrual (53 patients over 8 years). Thirty-nine patients had hypoxic tumours, of whom 19 received dose-escalation. The authors reported a non-significant improvement of 25% in local control for the dose escalation arm. Furthermore, of the patients treated with dose escalation, only a 2% mean elevation of radiotherapy dose was achieved, rather than the planned 10%. This trial highlights the difficulties of carrying out large-scale prospective imaging trials with [^18^F]FMISO. One of the reasons given for poor recruitment was scanner and tracer availability. In addition, the modest 2% dose escalation is much smaller than the 10% frequently quoted in planning studies and highlights the need to use real life patient data.

## Conclusion

Hypoxia PET imaging has been proposed for many years as a potential method to specifically target hypoxic tumour regions with higher doses of radiotherapy in order to improve outcomes. The results of our review challenge this notion. The answers to the four key questions posed in this review are given below:

1) Currently there is no clinically deliverable surrogate biomarker to predict for the [^18^F]FMISO hypoxic phenotype that would enable patient selection for [^18^F]FMISO imaging. This is a significant barrier as it is unlikely that [^18^F]FMISO PET scans can be routinely offered to all locally advanced HNC patients due to both availability and cost. The large number of studies trying to correlate [^18^F]FMISO PET with other hypoxia biomarkers highlights the need for a ‘proxy’ biomarker. As previously discussed, hypoxia is an umbrella term that refers to different biological processes on different assays, i.e., intracellular hypoxia versus interstitial and blood hypoxia, or acute versus chronic hypoxia. As such, we are far from identifying a hypoxia biomarker that would enable selection of patients for [^18^F]FMISO PET imaging.
2) The results of [^18^F]FMISO PET imaging are not repeatable and the location of uptake may vary during treatment. This highlights that hypoxia is a dynamic phenomenon which, in turn, renders it difficult, or even impossible, to reproduce with current tracers. As such, a single snapshot [^18^F]FMISO PET image, even if it faithfully maps hypoxia present at the time, does not provide all the information required for radiotherapy dose modification. Future work with novel tracers with shorter half-life and faster pharmacokinetics may provide additional insight.
3) Tumour recurrences do not necessarily occur within the pretreatment hypoxic volume on [^18^F]FMISO PET. This suggests that it may not be sufficient to escalate the radiotherapy dose to the hypoxic volume alone as radioresistant clones may not be fully targeted. Dose escalation to the whole tumour volume might be more appropriate.
4) Dose modification studies published thus far lend no credence to the *efficacy* of dose painting or modification based on [^18^F]FMISO PET imaging. The de-escalation studies have not proved that it was the absence of hypoxia on [^18^F]FMISO PET which made treatment de-escalation safe. Furthermore, the single dose escalation study illustrated the challenges of carrying out [^18^F]FMISO PET in a large-scale trial and the difficulties for dose escalation in real-world patients.

In summary, this review does not lend support to the notion of [^18^F]FMISO PET being used as a guide to adapt radiotherapy dose in HNC patients. Cost and access to [^18^F]FMISO PET are a real issue, especially for patients on a curative chemoradiotherapy treatment pathway for whom additional investigations need to be easily accessible and carried out promptly to avoid treatment delay. Ideally, a ‘proxy’ biomarker of hypoxia is needed, but this may be difficult to establish. The concept of dose escalation to the hypoxic volume specifically is not yet proven, and given that hypoxia is variable, dose escalation to a hypoxic region cannot be supported using a single scan. Hypoxia is a difficult phenomenon to identify and measure as it varies spatially within the same tumour microenviroment and is a dynamic process which can change acutely (e.g. secondary to variations in blood flow), or chronically, due to changes in oxygen demand from tissues. The studies included in this review were based on HNC but the findings could potentially be extrapolated to other tumour sites as many of the issues mentioned are related to the biology of hypoxia and the different assays used to measure it.
